# Ceramide signaling in immunity: a molecular perspective

**DOI:** 10.1186/s12944-025-02642-2

**Published:** 2025-07-01

**Authors:** Himani Thakkar, Vinnyfred Vincent, Bhagirath Chaurasia

**Affiliations:** Division of Endocrinology, Department of Internal Medicine, Fraternal Order of Eagles Diabetes Research Center, University of Iowa, Iowa City, IA USA

**Keywords:** Ceramides, Sphingolipids, Dyslipidemia, Inflammation, Immune cells, Signaling

## Abstract

Ceramides are bioactive lipids that play a crucial role in cellular signaling and structural integrity (Nat Rev Mol Cell Biol 19:175-191, 2018). As members of the sphingolipid family, ceramides consist of a sphingoid base attached to a fatty acid (Annu Rev Biophys 47:633-654, 2018). Their unique structure confers both hydrophobic and amphipathic properties, enabling them to organize into membrane microdomains that influence cellular dynamics (Annu Rev Biophys 47:633-654, 2018). In recent years, ceramides have garnered attention for their role in modulating a range of cellular and organismal functions. Unlike other lipids that primarily serve structural roles, ceramides act as bioactive lipids in key signaling pathways, mediating stress responses such as inflammation, oxidative stress, growth inhibition, metabolism, autophagy, and apoptosis (J Lipid Res 60:913-918, 2019). Their regulatory effects are particularly important in immune cells, where ceramides can influence cell fate, modulate cellular metabolism, affect cytokine production, and dictate responses to external stimuli (Nature 510:58-67, 2014). Since ceramides maintain a dynamic equilibrium with other sphingolipids within a cell, understanding their role in immune cells in isolation provides only a partial perspective. Nevertheless, as a bioactive lipid and the central precursor of other sphingolipids, ceramides play a pivotal role in immune cells, deserving focused attention.

## Highlights

Highlights of ceramides roles in immune cells:

• Immune cell activation: Ceramide modulate the activation of immune cells, influencing their cytotoxic activity and cytokine secretion (Cell Death Dis 6:e1828, 2015).

• Immune cell trafficking: Ceramide regulate the migration of immune cells to sites of inflammation by influencing chemokine receptors and cell adhesion molecules (Exp Mol Med 55:1110-1130, 2023).

• Inflammatory response:Ceramides influence inflammatory responses by modulating the production of inflammatory cytokines and chemokines, impacting the activity of immune cells (Sci Rep 11:8259, 2021).

• Cell death regulation: Ceramide is a key mediator of programmed cell death (apoptosis) in immune cells, triggering cell death pathways when its levels are elevated (Adv Med Sci 68:417-425, 2023).

• Interaction with other signaling pathways: Ceramide interact with other signaling pathways (e.g., Akt and MAPK), affecting cell survival, proliferation, and differentiation (J Biol Chem 275:13330-13335, 2000).

## Ceramide biosynthesis

Endogenous ceramides are comprised of a sphingoid base with 18 carbons, a 4,5-trans double bond, and an acyl chain that ranges from 12 to greater than 26 carbons in length [10]. The acyl chain and the sphingoid base can have varying degrees of unsaturation. However, emerging evidence indicates that sphingolipids with sphingosine bases of varying carbon lengths—such as d16, d17, and d20—are also biosynthesized under specific physiological or pathological conditions [11–13].

**Fig. 1:**
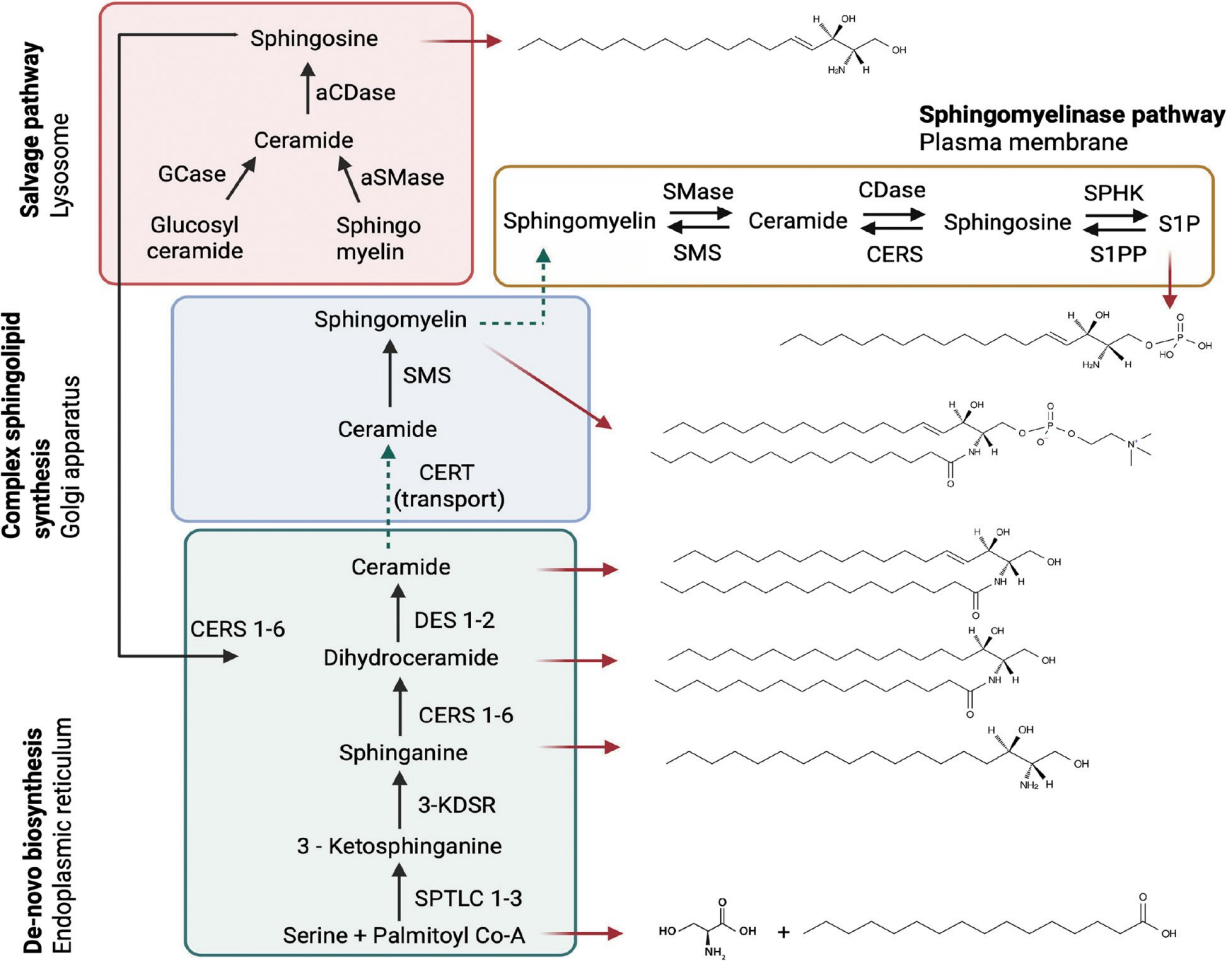
Sphingolipid metabolic pathways, organelles, and key sphingolipid structures. Schematic depicting the key pathways and enzymes involved in ceramide biosynthesis and degradation. Abbreviations: aSMase, acid sphingomyelinase; CDase, ceramidase; aCDase, acid ceramidase; CERS, ceramide synthase; CERT, ceramide transporter; Co-A, coenzyme A; DES, dihydroceramide desaturase; GCS, glucosylceramide synthase; GCase, glucosylceramidase; KDSR, 3-ketodihydrosphingosine reductase; SPTLC, serine palmitoyltransferase; SMS, sphingomyelin synthase; SPHK, sphingosine kinase; SMase, sphingomyelinase; S1PP, sphingosine 1-phosphate phosphatase

The sphingolipid family encompasses over 4,000 distinct lipid species that play key roles in cellular integrity and function. Ceramides lacking the 4,5-trans double bond are called dihydroceramides and are an important intermediate for de novo ceramide biosynthesis. Ceramides are central precursors for critical cellular sphingolipids, including sphingomyelins and gangliosides which are formed by the addition of chemical groups such as the phosphocholine and carbohydrates respectively, to carbon 1 (Fig. 1). Unlike other lipids, ceramides are not appreciably absorbed from the diet but are either synthesized de novo from saturated fats and proteins or regenerated from other sphingolipids—both processess that are finely regulated by specific enzymes that control ceramide production in response to physiological cues [14].

De novo ceramide biosynthesis takes place on the cytosolic surface of the endoplasmic reticulum (ER) in a multi-step cascade. The first and rate-limiting step is catalyzed by serine palmitoyltransferase (SPT), which typically condenses palmitoyl-CoA (C_16:0_) with L-serine to form 3-ketodihydrosphingosine, the precursor to sphinganine (d18:0) [1, 15]. However, SPT exhibits notable substrate flexibility, allowing for the incorporation of alternative acyl-CoAs such as myristoyl-CoA (C_14:0_) or stearoyl-CoA (C_18:0_), resulting in the synthesis of non-canonical sphingoid bases with 16 and 20 carbon atoms, respectively [16, 17]. These atypical sphingoid bases are less abundant than the canonical C18-sphinganine but are increasingly recognized for their physiological and pathological relevance [18, 19]. In addition to acyl-CoA variability of the sphingoid base, SPT can also utilize alternative amino acid substrates, such as alanine and glycine, although with reduced catalytic efficiency [20]. These substitutions yield atypical 1-deoxysphingolipids (deoxySLs), which lack the critical C1-hydroxyl group found in typical sphingoid bases. This structural difference renders deoxySLs unable to form complex sphingolipids or be degraded via canonical catabolic pathways, often resulting in their accumulation and are associated in the development of neurotoxicity and metabolic disorders [21–23].

SPT is an evolutionarily conserved enzyme and functions as a homodimer in prokaryotes and as a heterodimer in eukaryotes, primarily composed of SPTLC1 paired with either SPTLC2 or SPTLC3 [24]. Among these, SPTLC2 harbors the catalytic activity. The specificity and activity of the SPT complex are further modulated by small subunits ssSPTa and ssSPTb, which influence the enzyme’s acyl-CoA chain length preference for the synthesis of sphingoid base [17]. Additionally, ORMDL proteins (ORMDL1-3), integral ER membrane proteins, form a feedback loop to negatively regulate SPT activity in response to intracellular sphingolipid levels [25]. Together, these components—SPTLC1/2 or SPTLC1/3, ssSPTs, and ORMDLs—assemble into a higher-order regulatory complex that integrates cellular lipid status to finely tune sphingolipid biosynthesis, thereby maintaining membrane integrity and lipid homeostasis [26].

The reaction catalyzed by SPT yields 3-ketosphinganine, which is rapidly reduced to sphinganine and subsequently acylated by ceramide synthase enzymes (CERS1-6) to form dihydroceramides with variable acyl chain lengths [27]. Dihydroceramide desaturases then convert these to ceramides by introducing a 4,5-trans double bond, imparting ceramides its unique biophysical properties [14]. Ceramides are subsequently transported to the Golgi apparatus by ceramide transporter (CERT), where head groups such as phosphocholine or sugar moieties are added to form complex sphingolipids like sphingomyelins. CERT specifically mediates non-vesicular transport of ceramide from the endoplasmic reticulum (ER) to the Golgi, where sphingomyelin synthase catalyzes the transfer of a phosphocholine group from phosphatidylcholine (PC) to ceramide, producing sphingomyelin (SM) [28] (Fig. 1). CERT is capable of efficiently transferring various ceramide molecular species that naturally exist in mammalian cells, including those with C_14_–C_20_ saturated acyl chains, as well as C_16_-dihydroceramide and C_16_-phytoceramide [29]. Structurally, CERT is a cytosolic lipid transfer protein composed of three key functional domains: a pleckstrin homology (PH) domain, which targets the Golgi by binding phosphatidylinositol 4-phosphate (PI4P), an FFAT motif, which anchors CERT to the ER via interaction with VAMP-associated proteins (VAP), and a START domain, which binds and transfers ceramide [30, 31].This modular domain architecture enables CERT to bridge the ER and Golgi membranes and efficiently transport ceramide [14].

Ceramides can be regenerated through the breakdown of sphingomyelin by sphingomyelinases (sphingomyelin hydrolysis), which liberate ceramides while releasing the choline head group [32]. Sphingomyelinases (SMases) are classified into acidic (ASMase), neutral (NSMase), and alkaline (Alk-SMase) based on optimal pH and subcellular localization [1]. ASMase (encoded by *Smpd1*) primarily functions in lysosomes, while NSMases, including NSMase-1 and NSMase-2 (encoded by *Smpd2* and *Smpd3*, respectively), are located in the plasma membrane [33]. NSMase-2, in particular, is regulated by inflammatory cytokines like TNF-α and IL-1β and is involved in cellular stress responses [34]. Alk-SMase (encoded by *Enpp7*), primarily acts in the gastrointestinal tract to hydrolyze dietary SM [35]. Depending on the SMase type activated, ceramide is rapidly generated at specific intracellular sites, influencing distinct signaling pathways [33]. Ceramides can be regenerated through a process known as the salvage pathway [1]. Constitutive degradation of sphingolipids and glycosphingolipids occurs in the acidic environments of late endosomes and lysosomes, where they are broken down into ceramides. Ceramides are then further hydrolyzed by acid ceramidase into sphingosine and free fatty acids, both of which can exit the lysosome. The released sphingosine can be recycled through the salvage pathway, where ceramide synthases converts it back into ceramide [36] (Fig. 1).

Ceramides can be degraded by ceramidases [37]. This occurs primarily in lysosomes [38]. In mammals, five ceramidases have been identified, each with a distinct optimal pH: acid ceramidase (*Asah1*/AC), neutral ceramidase (*Asah2*/NC), and three alkaline ceramidases (*Acer1, Acer2,* and *Acer3*). Among them, the lysosome-localized *Asah1* is the physiologically most important [38]. In contrast, neutral ceramidase is primarily found at the plasma membrane, where it contributes to extracellular ceramide metabolism, while alkaline ceramidases are distributed across the endoplasmic reticulum and Golgi apparatus, involved in various cellular processes such as differentiation, apoptosis, and autophagy [39, 40]. Sphingosine produced by ceramides can then be converted to sphingosine-1-phosphate (S1P), a bioactive lipid with roles opposing those of ceramides, particularly in cell survival and proliferation pathways [41]. S1P may either be recycled back to ceramides via the salvage pathway, secreted out of the cell for autocrine or paracrine binding with its receptors (S1PR1-5), or irreversibly broken down by S1P lyase, diverting the molecules out of the sphingolipid pathway (Fig. 1). The activity of the ceramide biosynthetic pathways determines the sphingolipid levels in cells, and they are regulated in cell and context-specific manners.

### Biophysical properties of ceramides that regulates its function

Ceramides, as a bioactive lipids significantly influence cellular processes, including membrane dynamics and signaling pathways, the effects that could be influenced by its biophysical properties, some of which are discussed here. The hydrophobic nature of ceramides and their ability to form strong hydrogen bonds lead to the formation of gel-like domains within lipid bilayers, reducing membrane fluidity [42]. Ceramide generation or enrichment within the membrane, alters membrane order and induces phase separation, forming gel domains in the membrane [43]. These domains cause significant changes in membrane structure and function, including vesicle aggregation, fusion, and the release of intravesicular contents [44–46]. Moreover, ceramide induces mitochondrial-mediated apoptosis by regulating membrane permeability and pore formation that allows the release of small proteins from the mitochondria [47, 48].

Biophysical studies on both model and cellular membranes have revealed that elevated ceramide content in lipid rafts increases membrane order and stability, making these regions more mechanically rigid [49, 50]. This change enhances the interaction between proteins and lipids, facilitating the formation of signaling complexes. Moreover, the acyl chain length of ceramide influences its biophysical effects, with long-chain ceramides leading to less tightly packed gel domains compared to shorter-chain variants, affecting membrane properties and cell signaling outcomes [51].

Ceramide’s biophysical effects are highly dependent on lipid composition. In membranes rich in cholesterol, ceramide's ability to form gel domains is inhibited, while in cholesterol-poor membranes, ceramide-enriched domains can form more readily [52, 53]. This ability to modulate membrane properties is crucial in regulating cell signaling and receptor activity. For example, ceramide’s effects on membrane organization can entrap receptors and signaling molecules, potentially activating or inhibiting specific pathways [54]. Studies show that ceramide can alter membrane protein dynamics, such as the lateral diffusion of GPI-anchored proteins, by stabilizing their interactions in ceramide-rich domains [2, 55].

### Mechanisms by which ceramides modulate immune cell functions

Ceramides are known to modulate immune cell functions mainly through two mechanisms [3].

### Through modulation of membrane dynamics

Ceramides have distinct physical properties, which play a crucial role in modulating membrane dynamics [2]. One of the key functions of ceramides is their ability to form ceramide-rich platforms (CRPs) within the lipid bilayer, influencing the organization of membrane proteins and facilitating various cellular processes, including immune response and cell death [56]. These CRPs promote receptor clustering and protein recruitment, which are essential for cellular signaling [57]. For example, ceramide-induced clustering of the Fas cell surface death receptor (Fas receptor; CD95) at the plasma membrane is critical for the formation of the death-inducing signaling complex and the subsequent activation of caspases, emphasizing ceramides’ involvement in regulating cell death pathways [51, 58–60].

In addition to their role in receptor clustering, as mentioned above, ceramides also affect membrane fluidity and permeability. The length and saturation of ceramide acyl chains influence membrane fluidity, which in turn regulates processes such as cell migration. Moreover, ceramides can impact membrane permeabilization by forming channels in organellar membranes, including those of mitochondria and lysosomes, contributing to cellular homeostasis [61–64]. Together, these effects on membrane properties highlight ceramides as key modulator of cellular function and signaling.

### Through ceramide-binding proteins

Ceramides influence cell functions by binding to ceramide-binding proteins (CBPs) located in both the membrane and intracellular compartments. Some of the most studied CBPs include protein phosphatases (PP1 and PP2A), referred to as ceramide-activated protein phosphatases (CAPPs), along with protein kinase C zeta (PKC-ζ) and cathepsin D [65]. CAPPs play essential roles in regulating processes like apoptosis, mitosis, glycogen metabolism, and insulin signaling, and are crucial for controlling Akt phosphorylation, which has implications in cancer and insulin resistance [66, 67].

In addition to these well-known CBPs, an expanding list of putative ceramide-binding proteins is being identified, which participate in a variety of cellular functions. Moreover, ceramides affect mitochondrial processes by interacting with proteins involved in mitophagy and electron transport. For instance, C_18_-ceramide in mitochondria has been shown to interact with LC3BII, anchoring autophagosomes to mitochondria to promote lethal mitophagy [8, 67], while C_16_-ceramide disrupts fatty acid oxidation and electron transport by inactivating complexes II and IV of the respiratory chain [68, 69]. C_16_-ceramide also binds with high affinity to the DNA-binding domain of p53 [70].

On the mitochondrial membrane, voltage-dependent anion channels (VDAC1 and VDAC2), especially VDAC2 – function as ceramide-binding proteins. The interaction of ceramide with VDAC2 increases mitochondrial membrane permeability, facilitating cytochrome c release and promoting intrinsic apoptosis, a mechanism particularly relevant in cancer cells [71, 72]. In addition, ceramide synthesized by *CerS6*, especially C_16:0-_ceramide, binds to mitochondrial fission factor (MFF), a key regulator of mitochondrial fragmentation. This interaction promotes mitochondrial fission, contributing to obesity-related insulin resistance by impairing mitochondrial function and disrupting energy metabolism. Notably, deficiency in either *CerS6* or MFF protects against fatty acid-induced mitochondrial fragmentation in vitro and mitigates obesity-induced mitochondrial dysfunction in vivo [73].

In the lysosomal and endosomal system, lysosome associated protein transmembrane 4B (LAPTM4B) regulates ceramide levels in late endosomes via a sphingolipid interaction motif and an acidic transmembrane residue that mediate ceramide binding [74]. This interaction modulates mammalian target of rapamycin complex 1 (mTORC1) signaling, linking sphingolipid metabolism to cell growth and autophagy [75, 76]. PAQR4, a member of the progestin and adipoQ receptor (PAQR) family, regulates adipocyte function and systemic metabolic health by mediating ceramide levels. It stabilizes ceramide synthases (CERS2 and CERS5), enhancing their activity and leading to increased ceramide accumulation. This accumulation impairs adipogenesis and triggers adipocyte de-differentiation, contributing to metabolic disorders [77]. Similarly, ER-localized translocating chain-associated membrane proteins (TRAM1 and TRAM2), have been shown to bind ceramide and its analogs, influencing protein translocation across the ER [78].

Moreover, ceramide mediates the interaction of cytosolic proteins, such as S-palmitoylated, acetylated α-tubulin, with cellular membranes by forming ceramide-rich platforms. These ceramide-rich platforms play a crucial role in stabilizing microtubules and supporting ciliogenesis [79].

Notably, the above mentioned ceramide-protein interactions were found in numerous cells types (e.g., cancer cells, muscle cells, adipocytes etc.) and influence their functions. Future studies are warranted to determine whether these interactions and their functions are conserved in immune cells.

## Role of ceramides in innate immune cells

### Macrophages

Macrophages are essential components of the innate immune system, responsible for phagocytosis, antigen presentation, and the production of pro-inflammatory cytokines. Macrophage activation is a dynamic and essential process that enables these versatile cells to adapt to various stimuli and perform diverse functions. Initially classified into two categories—M1 (pro-inflammatory) and M2 (anti-inflammatory)—macrophages can be classically activated (M1) by pro-inflammatory cytokines like interferon-γ (IFN-γ) and tumor necrosis factor (TNF-α), leading to enhanced inflammatory responses and pathogen clearance [80]. Alternatively, they can undergo alternative activation (M2) in response to anti-inflammatory signals such as IL-4 and IL-13, promoting tissue repair and immune regulation [81, 82].

However, recent studies have shown that the M1/M2 dichotomy oversimplifies macrophage activation, as phenotypes are highly plastic and influenced by a wide range of intrinsic and extrinsic factors, including cytokines, chemokines, pathogen-associated molecular patterns (PAMPs), and pathogen recognition through toll-like receptors (TLRs) [83]. This results in a broad spectrum of macrophage phenotypes that vary in gene expression and cytokine production, complicating the identification of distinct M1 and M2 markers [84]. Macrophages can shift between intermediate or hybrid phenotypes—such as M4 and Mhem in atherosclerosis, and regulatory macrophages (MRs) in immune tolerance—further emphasizing the complexity of macrophage activation [85–87]. These phenotypes reflect the spatiotemporal nature of macrophage polarization, which is influenced by the microenvironment and can be reversible in response to changing conditions. The remarkable plasticity of macrophages allows them to fulfill critical roles in both homeostasis and disease, underscoring the need to understand macrophage activation as a continuum rather than a fixed classification [80, 88].

**Fig. 2:**
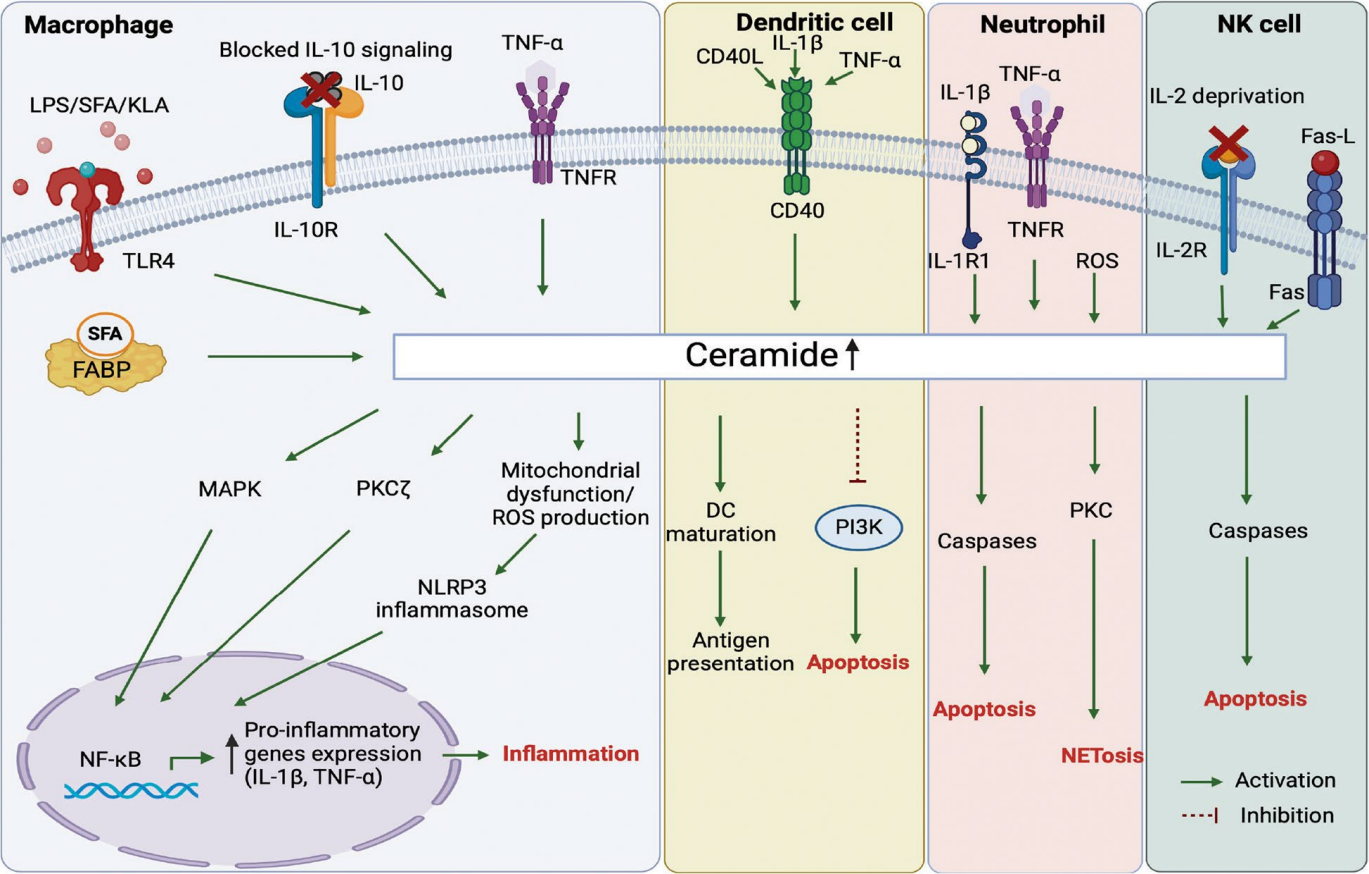
Ceramide signaling in innate immune cells. Summary of the pathways involved in promoting ceramide biosynthesis and its molecular and functional effects in innate immune cell subsets (macrophages, dendritic cells, neutrophils) and NK cells. Abbreviations: FABP, Fatty Acid- Binding Protein; IL-10, Interleukin 10; IL-10R, Interleukin receptor 10; KLA, Kdo2-lipid A; LPS, Lipopolysaccharide; MAPK, Mitogen-activated protein kinase; NETosis, Neutrophil Extracellular Trap formation; NF-kB, Nuclear Factor kappa-light-chain-enhancer of activated B cells; NLRP3, NOD-like receptor family pyrin domain-containing 3; PKC, Protein Kinase C; PI3K, Phosphoinositide 3-kinase; ROS, Reactive oxygen species; SFA, Saturated fatty acid; TLR4, Toll-like receptor 4; TNF, Tumor Necrosis Factor; TNFR, Tumor Necrosis Factor Receptor; TNF-α, Tumor necrosis factor-alpha

In macrophages, ceramides are generated in response to various stimuli, including PAMPs, pro-inflammatory cytokines, and nutrient cues [7, 89–92]. Activation of macrophages with Kdo2-lipid A (KLA), a specific (TLR4 ligand, significantly increases sphingolipid content, particularly ceramides, leading to larger cells with numerous autophagosomes crucial for autophagy [93]. Specifically, saturated fatty acids (SFA), which are components of Western diets, mimic pathogen-associated molecular patterns that regulate innate immune function, contributing to hyper-responsiveness to inflammatory stimuli. Exposure of macrophages to SFA, particularly palmitic acid (PA), significantly enhances the inflammatory responses of monocytes and macrophages to lipopolysaccharide (LPS) through a novel, TLR4-independent mechanism. This amplification occurs due to the activation of the ER stress-induced IRE1α pathway, increasing the expression of SPT [94]. Increased ceramide synthesis activates the inflammatory signaling pathways involving PKC-ζ and mitogen-activated protein kinase (MAPK) [95] leading to enhanced production of pro-inflammatory cytokines like IL-1β and TNF-α [90, 92]. This effect appears to be specific to saturated fatty acids, as unsaturated fatty acids either suppress inflammatory responses or have no significant effect [96]. Interestingly, IL-10, a crucial anti-inflammatory cytokine that suppresses macrophage activation, was recently found to reduce inflammation by potently inhibiting ceramide biosynthesis [97]. Moreover, macrophages lacking ASMase and reduced ceramide synthesis exhibit partial resistance to apoptosis during growth factor withdrawal, highlighting the importance of both ASMase activity and ceramides in apoptotic processes (Fig. 2) [89].

SFAs-induced ceramide biosynthesis also activates the NOD-like receptor family pyrin domain-containing 3 (NLRP3) inflammasome via activation of AMP-activated protein kinase (AMPK) and reactive oxygen species (ROS) production, leading to enhanced IL-1β secretion, which impairs insulin signaling in adipose tissue [98, 99]. Furthermore, adipose fatty acid-binding protein (A-FABP) in macrophages also induces ceramide production in macrophages in the presence of excess SFA and causes cell death [100]. This process contributes to chronic inflammation associated with obesity and metabolic disorders. Interestingly, while ceramide production through the enzyme SPT was hypothesized to be critical for this process, two independent studies revealed that myeloid cell-specific deletion of *Sptlc2* does not significantly alter macrophage responses or insulin resistance [101, 102].

Ceramides also influence macrophage polarization, which refers to the ability of macrophages to adopt to different functional states based on environmental cues. Ceramide treatment has been shown to promote the differentiation of macrophages into a pro-inflammatory M1 phenotype by increasing the expression of M1 marker CD68 and suppressing the expression of M2 markers [103, 104]. Sun et al., identified elevated ceramide accumulation in M1 macrophages, leading to increased hepatocyte apoptosis and liver dysfunction, while M2 macrophages promote sphingosine-1-phosphate generation, aiding hepatocyte proliferation and recovery [105].

Moreover, elevated ceramide production by treatment with long-chain saturated fatty acids like palmitic acid, promotes pro-inflammatory M1 macrophage polarization by inhibiting peroxisome proliferator-activated receptor (PPARγ) [106]. In contrast, monounsaturated fats (MUFAs), such as oleate activate PPARγ and support an anti-inflammatory M2 phenotype [107]. Additionally, distinct macrophage subsets demonstrate varying lipid utilization patterns, suggesting that their polarization state influences responses to environmental fatty acids and inflammation [108].

### Dendritic cells

Dendritic cells (DCs) are key antigen-presenting cells (APCs) that play a crucial role in linking innate and adaptive immune responses [109]. Upon exposure to pathogens or inflammatory signals, ceramide levels increase in DCs, leading to enhanced expression of co-stimulatory molecules like CD80 and CD86 [110]. This upregulation is vital for effective T cell activation, as DCs provide essential signals for T cell priming. However, it has been shown that CD40L, TNF-α, and IL-1β, all inducers of DC maturation, also trigger ceramide accumulation in DCs (Fig. 2). This accumulation impairs the cells'ability to capture and process antigens. For example, exogenous C_2_-ceramide treatment inhibits both macropinocytosis and receptor-mediated endocytosis in DCs, significantly reducing their capacity to present soluble antigens to T cells [111]. Furthermore, ceramide accumulation—following inhibition of acid ceramidase, disrupts major histocompatibility complex (MHC) class II-mediated antigen presentation, leading to reduced stimulation of CD4^+^ T cells and diminished cytokine production (e.g., IL-2), and impairing optimal immune responses [112].

While DCs are generally resistant to FasL-mediated apoptosis, a feature essential for their role in immune activation, ceramide accumulation disrupts this resistance, promoting cell death [113]. Tumor-derived ceramide species (such as C_16_, C_24:1_, and C_24:0_) have also been shown to induce apoptosis in DCs by inhibiting survival pathways like phosphoinositide 3-kinase/protein kinase B (PI3K/Akt) and extracellular signal-regulated kinase (ERK) (Fig. 2) [9, 114, 115]. Ceramides are also involved in regulating the migration of DCs to lymph nodes, where they present antigens to T cells [116]. The cellular outcomes of ceramide accumulation in DCs depend on factors such as its location, the extent of accumulation, and the timing of its production. The intricate balance between the enzymes of the sphingolipid synthesis and catabolism pathways plays a critical role in regulating DC lifespan, thereby influencing immune responses [117].

In contrast to the pro-apoptotic effects observed in tumor cells, a recent study identified a novel immune-stimulatory role for a synthetic ceramide analog, C_8_-ceramide, in viral infections. Specifically, C_8_-ceramide enhanced DC activation during lymphocytic choriomeningitis virus (LCMV) and influenza infections without inducing cell death. Instead, it promoted DC maturation, increasing the surface expression of MHC-I, MHC-II, and co-stimulatory markers, thereby enhancing T cell activation (Fig. 2). These findings suggest that C_8_-ceramide may offer a promising approach to modulate immune responses in viral infections without the adverse effects typically associated with ceramide-induced apoptosis [110].

Further investigation into the role of ceramides in DC maturation and immune responses reveals differences in lipid dynamics during tolerance-inducing versus inflammatory maturation of DCs. A key finding is the role of switch-associated protein 70 (SWAP-70), which regulates ceramide accumulation in DCs [118]. These findings underscore ceramide as a critical mediator of immune responses, particularly in DC activation and apoptosis, and highlight the importance of SWAP-70 in regulating lipid metabolism during immune activation. This research provides new insights into how lipid signaling influences immune responses and could inform therapeutic strategies targeting lipid pathways for immune modulation.

### Neutrophils

Neutrophils, as key players in the immune response, have been shown to interact with ceramides in several ways that impact their function [119] (Fig. 2). Ceramides can be generated in neutrophils through various signaling pathways, including those activated by oxidative stress, infection, or inflammatory stimuli (Fig. 2). Once produced, ceramide can modulate multiple aspects of neutrophil biology, including migration, adhesion, and apoptosis [120]. Neutrophil functions such as extravasation, migration, cytokine production, superoxide generation, and neutrophil extracellular trap (NETosis) formation are essential for immune responses to extracellular pathogens (Fig. 2) [121]. Early research identified ceramides as critical regulators in these processes, linking them to TNF-α signaling [122]. However, subsequent research revealed that ceramides negatively regulate superoxide production. Specifically, C_2_-ceramide inhibited arachidonic acid-induced superoxide formation and respiratory bursts in N-formyl-methionyl-leucyl-phenylalanine (fMLP) – stimulated neutrophils [123]. Neutral sphingomyelinase-derived ceramides modulate Rac1/2 and RhoA GTPases, affecting neutrophil polarity and chemotactic responses [124]. These findings suggest that ceramides play a dual role, initially delaying superoxide production to facilitate migration and later regulating inflammatory responses.

Ceramides also mediate apoptosis in neutrophils through caspase activation, particularly involving C_16_- and C_24_-ceramide species (Fig. 2) [125]. Anti-apoptotic factors like granulocyte–macrophage colony-stimulating factor (GM-CSF) reduce ceramide levels to delay apoptosis. ASMase-generated ceramide is essential for early neutrophil apoptosis, with delayed apoptosis observed in ASMase-deficient mice [126]. Furthermore, ceramides contribute to antimicrobial responses, such as ROS-induced mitochondrial ceramide production during *Pseudomonas aeruginosa* infections, which triggers cytochrome c release and cell death [127].

Ceramides have been shown to play an anti-inflammatory role in neutrophils as well by interacting with the inhibitory receptor CD300f. Ceramide-CD300f interaction suppressed the release of neutrophil chemoattractants from *Escherichia coli*-stimulated mast cells and neutrophils. Disruption of ceramide-CD300f interactions leads to increased neutrophil infiltration and heightened inflammatory responses in sepsis, protecting CD300f knockout mice from sepsis-induced death [128]. Ceramide-CD300f binding inhibited lipopolysaccharide-induced skin inflammation as well [129]. These findings highlight ceramides' diverse regulatory effects in neutrophil biology.

Recent studies have highlighted the critical roles of IL-1β-induced ceramide synthesis and protein kinase C (PKC) isoforms in the regulation of NETosis. IL-1β triggers ceramide production in neutrophils, which in turn promotes NET formation, a process linked to the progression of diseases such as abdominal aortic aneurysms [130]. Concurrently, PKC isoforms, including PKCβ, PKCδ, and PKCζ, are essential for mediating oxidative burst, cell spreading, and NETosis [131] (Fig. 2). Ceramide synthesis may activate PKC [132], creating a feedback loop that enhances NETosis and contributes to chronic inflammation.

### Natural Killer cells

Natural Killer (NK) cells play a pivotal role in immune surveillance, particularly in the elimination of infected and tumor-transformed cells [133]. Recent studies have highlighted the importance of ceramide in regulating NK cell functions, including activation, cytokine production, and apoptosis [134, 135]. Similar to its role in other immune cells, ceramide acts as a key mediator of apoptosis signaling in NK cells, particularly following receptor-mediated activation, such as Fas ligation [134].

In contrast to their role in activating NK cells, ceramides have been implicated in regulating NK cell apoptosis. A study published in 1998 showed that ceramide plays a critical role in Fas-induced apoptosis in NK cells and that redox regulation influences both ceramide generation and downstream signaling events, including protein tyrosine dephosphorylation, to control NK cell apoptosis [134] (Fig. 2). Additionally, C_6_-ceramide nanoliposomes and 1-phenyl-2-palmitoylamino-3-morpholino-1-propanol (PPMP), an inhibitor of GCS was shown to have synergistic effects, leading to increased ceramide levels in leukemic NK cells, inducing mitochondrial dysfunction, and triggering apoptosis. The mechanism of cell death involved the mitochondrial intrinsic apoptosis pathway, as evidenced by mitochondrial membrane depolarization and decreased levels of anti-apoptotic proteins like survivin and myeloid cells leukemia (Mcl)−1, which are key survival factors in NK leukemia [136]. ASM-generated ceramide in IL-2 deprivation induces apoptosis in NK/T lymphoma cells via a novel apoptotic pathway where ceramide activates cathepsin B in lysosomes, leading to the degradation of x-linked inhibitor of apoptosis (XIAP), which subsequently facilitates the nuclear translocation of cleaved caspase-3 [137] (Fig. 2).

IL-2 is known to promote NK cell expansion, and it has been shown that it does so by inhibiting ceramide generation through the regulation of ASMase, glucosylceramide synthase (GCS), and sphingomyelin synthase (SMS) [138]. This process is linked to the activation of PI-3 kinase, which not only supports cell growth but also inhibits ceramide generation through the regulation of ASMase, GCS, and SMS [138]. The balance between ceramide-induced survival and cell death signals is critical for maintaining proper NK cell function. Interestingly, ceramide regulation is not limited to apoptosis but also extends to NK cell activation. For example, α-galactosylceramide (αGalCer) strongly activates NK cells and triggers cytokine production (e.g., IL-4, IFN-γ) [135, 139]. This activation of NK cells increases the resistance to allogeneic bone marrow transplantation.

In parallel with ceramide signaling, a recent study highlights the critical role of glycosphingolipid (GSL) metabolism in NK cell biology. Specifically, super-enhancer loci in NK cells were found to contain genes *Ugcg* and *B4galt5* that encodes for UDP-glucose ceramide glucosyltransferase or GCS and β−1,4-galactosyltransferase 5 respectively, the two enzymes essential for GSL biosynthesis [140]. The study further showed that glycosphingolipids are essential for NK cell survival and cytotoxic function. Moreover, authors found that inhibition of UGCG activity disrupts granzyme B localization and the formation of functional immunological synapses, leading to NK cell apoptosis [141].

## Role of ceramides in adaptive immune cells

### B cells

B cells are a crucial component of the adaptive immune system, primarily responsible for producing antibodies that help to fight infections. They originate in the bone marrow and undergo a series of developmental stages, including the rearrangement of their immunoglobulin (Ig) genes, which allow them to produce unique antibodies that recognize specific antigens [142]. Once matured, B cells circulate in the bloodstream and migrate to secondary lymphoid organs, such as the spleen and lymph nodes, where they encounter antigens [143]. B cells are activated when their B cell receptors (BCRs) bind to specific antigens, a process often assisted by helper T cells through CD40 ligation and cytokine release [144]. Upon activation, B cells differentiate into plasma cells, which secrete large quantities of antibodies, and memory B cells, which retain the ability to respond rapidly to future infections by the same pathogen [145]. In addition to antibody production, B cells also act as antigen-presenting cells (APCs), helping to initiate immune responses by presenting antigens to T cells [146]. The function of B cells is tightly regulated to prevent autoimmunity, with mechanisms in place to eliminate or inactivate self-reactive B cells during their development [147, 148]. However, dysregulation of B cell activity can lead to autoimmune diseases like systemic lupus erythematosus (SLE) and rheumatoid arthritis, or immunodeficiency disorders when B cell function is insufficient [149–151].

**Fig. 3:**
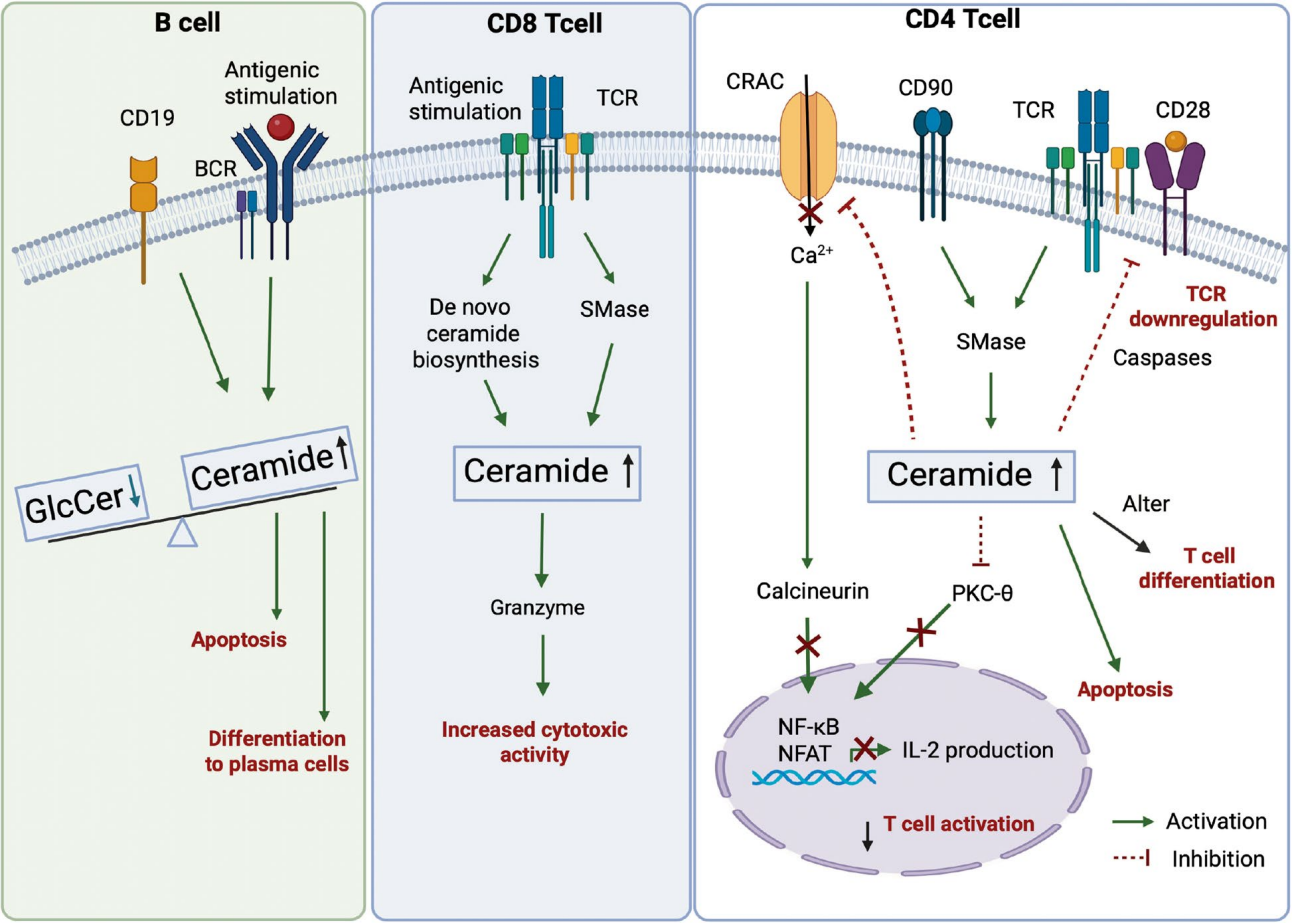
Ceramide signaling in adaptive immune cells. Overview of the pathways involved in inducing ceramide biosynthesis and its molecular and functional impacts on adaptive immune cell subsets (B cells, CD8 and CD4-Tcells). Abbreviations: BCR, B cell receptor; CRAC: Calcium Release-Activated Calcium channels; GlcCer, Glucosylceramide; NF-kB, Nuclear Factor kappa-light-chain-enhancer of activated B cells; NFAT, Nuclear Factor of Activated T cells; SMase, sphingomyelinase; TCR, T cell receptor

The studies highlights the multifaceted role of ceramide in regulating B cell fate, particularly in chronic lymphocytic leukemia (CLL) and other B cell types. Upon B-cell receptor (BCR) activation, CLL cells exhibit a metabolic shift—ceramide levels decrease while glucosylceramide increases, promoting cell survival and resistance to apoptosis (Fig. 3). This shift in the ceramide-to-glucosylceramide ratio is critical, as inhibiting glucosylceramide synthesis using UGCG inhibitors restores apoptosis, especially when combined with ceramide-inducing agents like ABT-737 [152]. In parallel, other studies show that BCR-triggered apoptosis involves a two-phase ceramide response: an early, caspase-independent rise in C_16_-ceramide leading to proteasomal activation and XIAP degradation, followed by caspase-dependent accumulation of C_24_-ceramide that amplifies apoptosis [153–155]. Additionally, long term exposure of ceramide levels suppress antigen-triggered calcium responses in B lymphocytes, while mature B cells like Burkitt lymphoma and marginal zone B cells show resistance [156]. In contrast to these detrimental effects, ceramide plays a crucial role in promoting B cell differentiation into plasma cells (PCs) during immune responses (Fig. 3). LPS activation induces metabolic changes, including increased ceramide biosynthesis, regulated by X-linked inhibitor of apopotosis protein (XBP-1) [157]. This process supports plasma cell differentiation, ER membrane expansion, and protein glycosylation for antibody production. Furthermore, ceramide promotes sphingolipid remodeling, particularly sphingomyelin synthesis, vital for membrane biogenesis and signaling. Together, these findings underscore the therapeutic potential of modulating ceramide metabolism in B cells, especially for targeting malignant B cell survival and promoting efficient plasma cell differentiation in immune responses.

### CD4^+^ T cell lymphocytes

T cells are a central component of the adaptive immune system, responsible for recognizing and responding to various pathogens, tumors, and self-antigens [158]. They arise from hematopoietic stem cells in the bone marrow and mature in the thymus, where they undergo selection processes that ensure self-tolerance and functional competence [159]. Upon encountering antigens presented by major histocompatibility complex (MHC) molecules on APCs, T cells become activated and differentiate into distinct subtypes with specialized functions. The main subtypes of T cells include CD4^+^ helper T cells, CD8^+^ cytotoxic T cells, regulatory T cells (Treg), and memory T cells, each playing a pivotal role in immune defense and homeostasis [160]. T cells can initiate, sustain, or suppress the immune response. Initiation and enhancement of immune response are caused by the cytotoxic T cells and helper T cells, while the suppression of immune response is caused by the Treg cells [161].

### Ceramides in T cell differentiation

Numerous studies have implicated the requirement of sphingolipids in the differentiation, and function of CD4^+^ T cells, including Th1, Th2, Th17, and Treg subsets [162, 163]. Employing genome-scale metabolic modeling (GSMM) in human CD4^+^ T cells, Sen and colleagues highlighted the essential and selective role of sphingolipids, particularly ceramide and GSL pathways, in Th17 cell differentiation [164]. These findings were further corroborated by Kanno and colleagues, who found that indeed numerous ceramide and GSL species were markedly induced in in vitro differentiated Th17 and were crucial for their differentiation and proinflammatory cytokine expression (IL-17A) [164–166]. While these two studies indicated that sphingolipids selectively modulate Th17 cell differentiation, other studies have found that ceramides are required for Th1 and Treg cell differentiation in vitro [167, 168]. In contrast to these in vitro findings indicating the requirement of ceramides for specific T cell subset differentiation, other studies have found that ceramides impair the differentiation of naïve CD4^+^ T cells into Treg cells but no other CD4^+^ T cell subtypes [169]. In vivo, while inhibition of *Sptlc1* in CD4^+^ T cells impairs Th1 and Th17 differentiation [170], ablation of ASMase, which reduces ceramide levels, increases Treg cell differentiation [169]. Thus, while these studies provide support for the role of ceramides in modulating CD4^+^ T cell differentiation, there is a lack of clarity on how and which T cell subtypes are selectively modulated by ceramides and whether these effects are mediated by ceramides or other sphingolipids.

## Regulation of T cell activation by ceramides

### TCR modulation

T cell activation begins when the T cell receptors (TCRs), composed of TCR chains and CD3 molecules, recognize antigens presented by the MHC. T cells express either α-β or γ-δ TCR chains, with α-β T cells being the most common [171]. Co-receptors CD4 and CD8 further mediate antigen recognition by binding to MHC class II and class I molecules, respectively [172]. Full TCR activation requires co-stimulation via CD28, which binds CD80/86 on antigen-presenting cells, activating the PI3K pathway and phospholipase C-γ (PLC-γ) and downstream signaling [173–175]. This cascade involves the phosphorylation of zeta-chain-associated protein kinase 70 (ZAP-70) and the activation of linker for activation of T cells (LAT), which activates key transcription factors [e.g., nuclear factor-κB (NFκB), activator protein 1 (AP-1), signal transducer and activator of transcription 5 (STAT5), and nuclear factor of activated T cells (NFAT)], promoting T cell proliferation, differentiation, and survival [176]. TCR signaling occurs in lipid rafts, specialized membrane regions that concentrate signaling molecules into a supramolecular activation cluster (SMAC), ensuring efficient signal transduction [177, 178].

Ceramides play a pivotal role in regulating TCR signaling and modulating T cell fate, exhibiting both pro-apoptotic and regulatory effects [5, 179]. Ceramides are generated upon engagement of death receptors like Fas/CD95 [180] or exposure to stress/inflammatory stimuli through sphingomyelinase activation [181]. When TCR engages with APCs, it triggers a cascade of signaling events that rapidly induce ceramide production (Fig. 3) [182]. Ceramide has a dual role in regulating T cell fate, depending on the concentration and duration of the stimulus. High levels of ceramide, especially when generated during prolonged or strong Fas activation, trigger apoptosis in T cells [183]. This apoptosis is a key mechanism for eliminating exhausted effector T cells, thus maintaining immune homeostasis and preventing autoimmune diseases. Conversely, ceramide also exerts non-apoptotic effects, particularly at lower concentrations when treated for shorter durations. At lower concentrations, ceramide inhibits T cell activation without inducing cell death. This modulation occurs through ceramide's impact on membrane raft reorganization, ion channels, and phosphorylation events, which influences calcium signaling and TCR-mediated activation (Fig. 3) [184]. For example, short-term exposure to ceramide has been shown to protect cytotoxic T cells (CTLL-2; cytotoxic T lymphocyte cell line-2, clone of cytotoxic T cells derived from a C57BL/6 mouse) from apoptosis by inhibiting the degradation of Bcl-xL, a protein involved in cell survival [185].

In contrast, prolonged or higher ceramide concentrations, such as those induced by chronic Fas engagement, promote apoptosis in T cells through mechanisms like caspase activation [186]. Moreover, ceramide modulates various intracellular signaling pathways, such as the inhibition of protein kinases like PKC-θ (protein kinase C-theta). Ceramide’s inhibition of PKC-θ disrupts NF-κB activation, leading to reduced IL-2 production which negatively regulates T cell activation [187] (Fig. 3). In addition, increased accumulation of ceramides in the plasma membranes interferes with TCR nanoclustering in CD4^+^ T cells [188] (Fig. 3). Moreover, sphingomyelinase-mediated ceramide enrichment in the T cell plasma membrane disrupts C-X-C chemokine receptor type 4 (CXCR4) receptor dynamics, impairing the T cell's ability to respond to chemokine gradients and hindering T cell migration, the critical processes in T cell recruitment during autoimmune responses and cancer metastasis [189].

### Calcium signaling

Calcium (Ca^2+^) signaling plays a critical and multifaceted role in T cell activation, differentiation, and immune responses [190]. Upon T cell receptor engagement, intracellular Ca^2+^ stores are mobilized through store-operated calcium enty mechanism, predominantly involving calcium release-activated calcium (CRAC) channels, composed of Orai1/2 proteins and Stim1 proteins [191, 192]. This Ca^2+^ influx is essential for various downstream signaling pathways, most notably the activation of calcineurin, a protein phosphatase that dephosphorylates the transcription factor NFAT [193]. This dephosphorylation allows NFAT to translocate into the nucleus and promote the transcription of genes like IL-2, a key cytokine for T cell proliferation and survival (Fig. 3). Membrane composition critically modulates calcium signaling efficiency. For example, the unsaturated fatty acid oleic acid enhances calcium flux upon CD3/CD28 stimulation in CD4⁺ T cells by incorporating into membrane lipids, suggesting that lipid membrane fluidity and structure play a role in optimizing Ca^2+^^2^⁺ channel function and signal transduction [194]. In contrast, ceramide exerts an inhibitory influence on Ca^2^^+^ signaling in T cells [2]. By altering membrane biophysical properties, ceramide can disrupt the function of CRAC channels, thereby blocking Ca^2^⁺ influx (Fig. 3). This suppresses the activation of calcineurin and NFAT, ultimately downregulating IL-2 production and impairing T cell activation (Fig. 3). Thus, ceramide dampens immune responses not only through the induction of apoptosis but also by directly antagonizing calcium-dependent signaling pathways [180, 195, 196].

## Role of ceramide species in T cells

TCR stimulation in activated T helper cells show a significant increase in ceramide production, particularly through the upregulation of ceramide synthases, *CerS2* and *CerS5* enzymes, which produce long-chain (C_22_–C_24_) and short-chain (C_14_–C_16_) ceramide species respectively [165]. This shift enhances glycosphingolipid production and supports TCR signaling, highlighting a potential regulatory role for ceramide species in immune activation [165]. However, in mice lacking *CerS2*, Th2 differentiation from naïve CD4^+^ T cells is reduced, while Th17 differentiation is enhanced. This shift is partly due to changes in TCR signaling and membrane properties, as *CerS2* influences lipid rafts and TCR nanoclustering [197].

*CerS4* is an essential enzyme in the synthesis of long-chain ceramides (C_18_-C_20_), and its role extends beyond lipid metabolism to regulate critical immune responses [198]. *CerS4* plays a critical role in T cell function and immune responses in colitis and colitis-associated cancer. In T cells, *CerS4* regulates several key pathways, including TGFβ signaling, NF-κB activation, and cytokine production [199]. *CerS4* deficiency in T cells impairs T cell proliferation, and immune resolution, and enhances tumor progression in models of colitis and cancer, respectively [200, 201].

*CerS5* and *CerS6* both contribute to the generation of short-chain ceramides, but their physiological effects differ. For example, *CerS5* and *CerS6* knockout mice show increased susceptibility to DSS-induced colitis and colitis-associated colon cancer [60, 202]. However, *CerS6* deficiency is linked to increased neutrophil infiltration, while *CerS5* deficiency leads to a reduction in CD3^+^ T cells, including CD4^+^, CD8^+^, and Treg subsets, in the colon, blood, and spleen. This reduction suggests that *CerS5* affects T cell migration and activation. Moreover, *CerS5* depletion in T cells impairs TCR signaling, reduces NF-κB activation, and impairs cytokine production (e.g., IFN-γ and IL-4) [202].

In inflammatory models, such as colitis and graft-vs-host disease (GVHD), *CerS6*-deficient T cells exhibit reduced inflammation, suggesting a protective effect [203]. *CerS6* contributes to T cell-driven inflammation by enhancing T cell proliferation and IFN-γ production. In contrast, pharmacological inhibition or genetic ablation of *CerS6* impairs TCR signaling in response to alloantigens, leading to reduced T cell responses in GVHD and colitis [204]. This effect is mediated through the regulation of TCR signaling in CD4^+^ T cells via the N-Ras/ERK pathway, which is critical for T cell activation, migration, and inflammatory responses [204]. In addition, *CerS6*-induced ceramide accumulation during aging has been shown to disrupt T cell antitumor activity [205]. Aging-related ceramide stress contributes to T cell dysfunction via mitophagy, limiting their antitumor and immune functions. Mechanistically, this is mediated by *CerS6*-induced ceramide accumulation, which inhibits protein kinase A (PKA) activation, and mitochondrial dysfunction, and induces mitophagy [205].

## CD8^+^ T cell lymphocytes

Ceramides regulate the cytotoxic functions of CD8^+^ T cells, which are essential for clearing infected or tumorigenic cells [206]. CD8^+^ T cells execute their cytotoxic response primarily through the secretion of perforin and granzymes, which induce apoptosis in target cells [207, 208]. The formation and function of the immunological synapse — the interface between the CD8^+^ T cell and its target — is critical for this process, and ceramide plays a significant role in this dynamic. Ceramide levels influence TCR signaling in CD8^+^ T cells and are required to protect against viral infections (Fig. 3) [209]. Specifically, in AC-deficient mice, ceramide-enriched platforms in the immunological synapse were found to enhance TCR signaling, suggesting that ceramide plays a crucial role in modulating TCR signaling strength and improving CD8^+^ T cell function [206]. Additionally, ASMase deficiency in CD8^+^ T cells led to reduced granzyme B production, impaired cytotoxicity, and diminished tumor size/burden [206]. Moreover, ablation of *Sptlc2* impairs the metabolic fitness of CD8^+^ T cells to elicit protective T cell responses [209]. Conversely, elevated ceramide levels promoted increased T cell activation and improved granzyme B production in CD8^+^ T cells, enhancing their cytotoxic function and anti-tumoral immune response, which ultimately resulted in reduced tumor growth [104, 206, 210].

## Diseases associated with ceramide mediated immune cell dysfunction

Ceramide-mediated immune dysfunction in chronic inflammatory diseases (obesity, diabetes mellitus, atherosclerosis, inflammatory bowel disease (IBD), etc.) and autoimmune diseases (systemic lupus erythematosus (SLE), multiple sclerosis (MS), Alzheimer’s disease (AD), etc.) are well established [4, 14, 211–215]. A common underlying factor for obesity-associated diseases like type 2 diabetes mellitus and atherosclerosis is chronic low-grade inflammation [216]. This inflammation is characterized by ceramide accumulation in adipose tissue and contributes to insulin resistance and activation of pro-inflammatory immune cells, which further exacerbates metabolic dysfunction. Moreover, ceramides activate NLRP3 inflammasomes in macrophages to induce cytokine secretion [99, 217]. Macrophage secretion of inflammatory cytokines such as TNF-α, IL-6, and IL-1β further induces ceramide production leading to a vicious cycle of inflammation in adipose tissue and subsequent insulin resistance [218]. In atherosclerosis, ceramide promotes the activation of endothelial cells and macrophages, leading to the formation of atherosclerotic plaques and vascular inflammation [219–221].

Recent studies suggest that ceramide metabolism is closely linked to the pathogenesis of inflammatory bowel disease, with both ceramide synthesis and catabolism influencing disease outcomes. Altered ceramide levels, due to changes in ceramide synthase isoforms, contribute to intestinal inflammation by disrupting the epithelial barrier and enhancing immune responses [60, 213, 222]. Increased ceramide levels in intestinal tissues can drive the differentiation of pro-inflammatory Th17 cells, leading to the secretion of cytokines such as IL-17 and IL-22, which intensify gut inflammation [223]. Furthermore, ceramides hinder the functions of regulatory T cells, impairing their ability to suppress effector T cell activity [224]. Ceramides also promote T cell migration to inflamed tissues and enhance cytokine secretion, further fueling the inflammatory response [225]. This dysregulation of T cell activity contributes to the chronic inflammation and immune dysfunction observed in IBD [226, 227]. For instance, myeloid-specific ceramidase knockouts protects against colitis by reducing neutrophil recruitment [203]. These findings suggest that ceramide regulation plays a vital role in immune cell activation, and inflammatory responses, presenting ceramide-related pathways as potential targets for therapeutic intervention in IBD.

Ceramide signaling has been implicated in other chronic inflammatory conditions including autoimmune diseases like SLE and MS where it triggers the activation of autoreactive immune cells, induces cytokine production, and enhances the inflammatory processes that drive tissue damage [228, 229]. In the central nervous system (CNS), elevated ceramide levels have been shown to activate the NF-κB signal transduction in microglia, shifting the glial response towards inflammation and prompting the release of TNF-α, IL-1β, and IL-6 from astrocytes, thereby inducing neuroinflammation in conditions like AD [230, 231]. In AD, ceramide is also implicated in neuroinflammation, neuronal apoptosis, and the formation of amyloid plaques, which are characteristic of the disease’s pathology [211, 232, 233]. Through these mechanisms, ceramide mediates immune dysfunction by modulating immune cell activation, promoting chronic inflammation, and contributing to tissue damage in these diseases. Although most of the studies on the role of ceramides in disease conditions have focused on the role of ceramides in instigating inflammation and immune cell activation, the evidence of ceramide dysregulation within the immune cells in these disease contexts is limited and warrants future attention.

## Therapeutic targeting of ceramide pathways in immune cells

Targeting ceramides in immune cells offers a compelling avenue for therapeutic intervention in a variety of diseases where immune dysfunction plays a pivotal role. Dysregulated immune cell ceramide signaling has been implicated in a spectrum of pathological conditions, including chronic inflammatory diseases like rheumatoid arthritis, autoimmune disorders such as lupus, metabolic diseases like obesity and diabetes, and cancer progression [99, 200, 214, 227, 232, 234]. By modulating immune cell ceramide levels, it may be possible to restore immune homeostasis and counteract disease progression.

Ceramide signaling in immune cells can impact their activation and function, offering the potential for immune modulation in cancer immunotherapy. Ceramide influences the immune response in the tumor microenvironment, and modulating ceramide levels enhances the effectiveness of immunotherapies [235]. Ceramide acts as a pro-inflammatory mediator by activating inflammatory signaling pathways, such as NF-κB signaling and cytokine production in macrophages. Thus, elevated ceramide levels can contribute to the amplification of the anti-cancer immune response. The frequencies and activities of different T cell subtypes in the tumor microenvironment regulate tumor progression, and ceramides can modulate this process. For example, exogenous C_2_-ceramide induces a strong anti-tumor response by increasing frequencies of cytotoxic CD8^+^ and IFN-γ-producing CD4^+^ T cells [104]. Using acid sphingomyelinase and acid ceramidase knockouts in CD4^+^ cells, which exhibit decreased or increased ceramide levels in T cells, respectively, it was revealed that increased ceramide concentrations improve the anti-tumoral T cell response during melanoma progression [206]. These findings highlight ceramides as important modulators of T cell function in the tumor microenvironment (TME). Treg cells also play a key role in modulating anti-cancer immune response. The infiltration of Treg cells into the tumor is considered a critical step during tumorigenesis, enhancing tumor progression [236, 237] and these cells are known to adapt to the tumor microenvironment by shifting their lipid metabolism – particularly through increased fatty acid uptake and oxidation, to sustain their suppressive function [238]. Although unclear, growing evidence suggests that ceramides play a role in the metabolic reprogramming essential for Treg survival and function within the TME [201, 239]. As ceramides emerge as key regulators of Treg biology, targeting their metabolic pathways presents a promising strategy to modulate Treg-mediated immunosuppression and enhance anti-tumor immune responses. Targeting immune cell ceramides is a promising strategy for treating autoimmune diseases as elevated ceramide levels within immune cells contribute to excessive inflammation by activating immune cell signaling pathways [6, 119, 234], making them a potential therapeutic target to modulate immune responses in autoimmune conditions such as rheumatoid arthritis, systemic lupus erythematosus, and multiple sclerosis.

Ceramide is required for maintaining intestinal homeostasis [213, 240]. Modulation of ceramides in neutrophils alters their migration to inflamed sites within the intestine. Depletion of acid ceramidase in myeloid cells, which increases ceramide levels, leads to decreased migration of neutrophils to the intestine and protects from experimental models of colitis and colitis-associated cancer development [241]. Interestingly, decreasing C_16_-ceramide species in T-lymphocytes was found to protect against experimental colitis development partly due to the decreased migratory capacity of the T cells [203]. Thus further opening the therapeutic opportunity to target specific ceramide species in inflammatory diseases.

## Future directions and open questions

While the role of ceramides in immune dysregulation across various disease contexts is well established, there remain significant gaps in understanding their precise role within specific immune cell subsets. Ceramides are known to modulate several critical immune processes, including immune cell activation, trafficking, inflammation, and programmed cell death [117, 234]. However, the cell-type-specific mechanisms by which ceramides influence these processes in T cells, macrophages, dendritic cells, neutrophils, and other immune populations are not fully elucidated.

The effects of different ceramide species—shaped by their acyl chain length, saturation, and subcellular localization—likely vary across immune cell types. To address this, future research employing single-cell lipidomics and metabolomics will aid in resolving the spatial and metabolic heterogeneity of ceramide signaling at the single-cell level. These approaches will help decipher how specific ceramide species regulate immune activation, cytokine production, migration to inflammatory sites, and cell death pathways. Complementary flux studies using stable isotope-labeled precursors can further illuminate the real-time dynamics of ceramide biosynthesis and turnover under different immunological conditions. To understand the molecular underpinnings of ceramide function, future studies could utilize genetic models, such as conditional knockout mice targeting ceramide synthases or sphingomyelinases in specific immune cell types. Moreover, CRISPR-Cas9-based gene editing in primary immune cells will provide a powerful strategy to dissect the roles of individual enzymes or regulatory nodes in the ceramide biosynthetic and signaling pathways.

Another major gap is the limited understanding of the temporal dynamics and subcellular compartmentalization of ceramide production during immune activation. This could be addressed through live-cell imaging techniques using ceramide-sensitive fluorescent probes, coupled with metabolic flux assays to track the synthesis and turnover of ceramides in different intracellular compartments. In addition, the composition and organization of ceramide-enriched membrane microdomains, which serve as signaling hubs for receptor clustering and downstream signaling—remain largely uncharacterized. Advanced proteomic tools, such as proximity labeling proteomics can be employed to identify the protein complexes assembled within these microdomains and define how ceramide scaffolds immune signaling at the membrane interface.

The role of ceramides in maintaining immune tolerance and preventing autoimmunity also remains poorly understood. Key questions include whether ceramide profiles are distinct in regulatory T cells and tolerogenic dendritic cells, and how alterations in ceramide metabolism may contribute to the breakdown of peripheral tolerance. Experimental autoimmune models, combined with selective ceramide modulation, can be used to investigate whether altering ceramide signaling restores immune homeostasis and prevents autoimmune responses.

Ceramides are emerging as key regulators of immune cell metabolism [115, 123, 136, 205], yet several fundamental questions remain unanswered. One major question is how ceramide impact metabolic pathways such as glycolysis, oxidative phosphorylation, and fatty acid oxidation across various immune cells. It is unclear whether these effects are cell-type specific or influenced by environmental cues. Another open question is how ceramides impair mitochondrial function and alter redox balance in immune cells. Do they directly affect mitochondrial integrity, or act via upstream regulators like PI3K/Akt or AMPK? The timing of ceramide accumulation is also not well understood—whether it serves as an early metabolic switch, or a marker of chronic stress remains to be determined. Further questions include how ceramides interact with other metabolic pathways, such as amino acid metabolism and mTOR signaling, and whether these interactions influence immune cell fate—activation, exhaustion, or memory formation. To explore these questions, future studies could combine lipidomics and metabolomics with genetic manipulation (e.g., ceramide synthase knockouts or pathway inhibitors), real-time metabolic assays (e.g., Seahorse), and isotope tracing. Single-cell multi-omics and in vivo models will be essential to define the context-specific metabolic roles of ceramides.

Finally, to translate these mechanistic insights into viable clinical applications, there is a need to develop precision-targeted therapeutic strategies. This includes the use of nanoparticle-based or antibody-conjugated liposomal delivery systems to modulate ceramide pathways in specific immune cell populations. Additionally, enzyme-specific modulators will ensure targeted manipulation of ceramide signaling with minimal off-target effects and systemic immune suppression.

Over the past decades, research has collectively shown that ceramides are tightly regulated and play a key role in modulating immune cell differentiation and function, thereby influencing the development of various inflammatory and potentially metabolic diseases. With the advent of novel tools and technologies, this field is now well-positioned to uncover how ceramides are regulated, trafficked between organelle and modulate metabolism within immune cells during their activation or suppression. Moreover, defining their cell-autonomous effects and underlying mechanisms could lay the foundation for developing new strategies to target ceramides within immune cells for the treatment of numerous diseases such as the inflammatory diseases, cancer and metabolic disorders.

## Data Availability

No datasets were generated or analysed during the current study.
